# New Insights in Abdominal Pain in Paroxysmal Nocturnal Hemoglobinuria (PNH): A MRI Study

**DOI:** 10.1371/journal.pone.0122832

**Published:** 2015-04-21

**Authors:** Francesco De Cobelli, Giulio Pezzetti, Sergio Margari, Antonio Esposito, Francesco Giganti, Giulia Agostini, Alessandro Del Maschio

**Affiliations:** Department of Radiology and Center for Experimental Imaging, San Raffaele Scientific Institute, Vita-Salute University, Milan, Italy; Wayne State University, UNITED STATES

## Abstract

**Introduction:**

Abdominal pain in PNH has never been investigated by *in-vivo* imaging studies. With MRI, we aimed to assess mesenteric vessels flow and small bowel wall perfusion to investigate the ischemic origin of abdominal pain.

**Materials and Methods:**

Six PNH patients with (AP) and six without (NOP) abdominal pain underwent MRI. In a blinded fashion, mean flow (MF, quantity of blood moving through a vessel within a second, in mL·s^-1^) and stroke volume (SV, volume of blood pumped out at each heart contraction, in mL) of Superior Mesenteric Vein (SMV) and Artery (SMA), areas under the curve at 60 (AUC_60_) and 90 seconds (AUC_90_) and K^trans^ were assessed by two operators.

**Results:**

Mean total perfusion and flow parameters were lower in AP than in NOP group. AUC_60_: 84.81 ± 11.75 vs. 131.73 ± 18.89 (*P *< 0.001); AUC_90_: 102.33 ± 14.16 vs. 152.58 ± 22.70 (*P *< 0.001); K^trans^: 0.0346 min^-1^ ± 0.0019 vs. 0.0521 ± 0.0015 (*P* = 0.093 duodenum, 0.009 jejunum/ileum). SMV: MF 4.67 ml/s ± 0.85 vs. 8.32 ± 2.14 (*P * = 0.002); SV 3.85 ml ± 0.76 vs. 6.55 ± 1.57 (*P* = 0.02). SMA: MF 6.95 ± 2.61 vs. 11.2 ± 2.32 (P = 0.07); SV 6.52 ± 2.19 vs. 8.78 ± 1.63 (*P* = 0.07). We found a significant correlation between MF and SV of SMV and AUC_60_ (MF:*ρ* = 0.88, *P *< 0.001; SV: *ρ* = 0.644, *P* = 0.024), AUC_90_ (MF: *ρ* = 0.874, *P *< 0.001; SV:*ρ* = 0.774, *P* = 0.003) and K^trans^ (MF:*ρ* = 0.734, *P* = 0.007; SV:*ρ* = 0.581, *P* = 0.047).

**Conclusions:**

Perfusion and flow MRI findings suggest that the impairment of small bowel blood supply is significantly associated with abdominal pain in PNH.

## Introduction

Paroxysmal Nocturnal Hemoglobinuria (PNH) is an acquired clonal disorder of hematopoietic stem cells due to somatic mutations in the PIG-A gene, with an early block in the synthesis of the glycosylphosphatidylinositol (GPI) anchor on the cell membrane [1].

There is a deficiency of GPI-anchored proteins CD55 and CD59, inhibiting factors of the complement: red blood cells are prone to complement-induced lysis, leading to persistent intravascular hemolysis with brisk exacerbations [1,2].

An increased susceptibility to thromboembolism is present [3]: complement directly initiates aggregation/activation of platelets and damage of endothelial cells [2,4,5].

In two retrospective studies of 220 and 460 PNH patients [6,7], the cumulative incidence rate of thromboembolic events was 30.7% with an incidence at the time of diagnosis of 7.2% [6] and a 10.2% relative risk of thrombosis [7].

The analysis of 195 PNH patients of three independent clinical studies and an open-label study [8–11] showed that 18.5% of the thromboembolic events regarded the mesenteric/splenic veins and 16.9% the portal vein [3,12]; thrombotic complications were the cause of death in the 44% of the patients [13].

Abdominal pain is one of the main causes of discomfort and disability in PNH and is present in approximately one-third of the patients at diagnosis [7]; it is associated with higher risk of thromboembolic events [14].

Albeit Magnetic Resonance Imaging (MRI) is considered the method of choice to assess vascular patency and parenchymal iron overload in PNH [15], the exact pathophysiology (i.e. the ischemic origin) has never been accurately investigated by *in-vivo* imaging studies.

Dynamic Contrast-Enhanced (DCE) MRI of the small bowel allows to investigate the mesenteric vessels flow, confirming the blood supply impairment in patients with chronic mesenteric ischemia [16–19].

Moreover, DCE-MRI parameters, area under the curve (AUC) and K^trans^, give important semi-quantitative/quantitative information about bowel wall perfusion. AUC is the area under the signal intensity curve from the time of contrast agent injection to usually 60 and 90 s [20].

K^trans^ represents the product of tissue blood flow and the incomplete first-pass extraction fraction of the contrast agent from the vascular system into the extravascular/extracellular space.

The extraction fraction is a function of the capillary permeability and blood flow [21].

To date, DCE-MRI functional assessment of small bowel microvascular perfusion and mesenteric flow analysis have never been performed in PNH.

Thus, in order to investigate the ischemic origin of abdominal pain, our purpose was to assess both the mesenteric vessels flow and small bowel wall perfusion using MRI.

## Materials and Methods

### Patients

This is a prospective study, approved by the San Raffaele Hospital Scientific Institute Ethics Board.

All patients provided written informed consent.

Between April 2012 and July 2013, twelve patients with PNH (7 women and 5 men, aged from 21 to 59 years), untreated or previously treated with Eculizumab (suspended more than three months before enrolment) were enrolled based on their clinical history of presence (AP) or absence (NOP) of abdominal pain.

Eculizumab is a humanized monoclonal antibody preventing the assembly of the membrane attack complex of the complement.

Exclusion criteria were:

- MRI contraindication;
- Renal failure (Glomerular filtration rate < 30ml·min^-1^);
- Intolerance to contrast medium or Scopolamine-butylbromide;
- Therapy with Eculizumab suspended less than three months before enrolment;
- History of drug/alcohol abuse;
- Any condition/ongoing medication able to induce abdominal pain.

The diagnosis of PNH was based on the granulocyte PNH clone in the peripheral blood assessed by flow cytometry analysis (at least 20% [22–24]) and on the increase of serum Lactate Dehydrogenase (LDH > 1.5 ULN).

All NOP patients enrolled in this study did not experience any abdominal pain attributable to PNH.

In the AP group, patients had more than 4 episodes of abdominal pain per year, with at least one episode in the quarter preceding the enrolment.

None of the AP patients had pain at the time of MRI scan.

The association of the pain with food assumption, the average duration of the single episodes of pain, the type of presentation, the association with hemolytic attacks (LDH levels), the average intensity (from 0 to 10) [25] and the concurrent antalgic, anti-spastic and anti-coagulant/anti-platelet therapies were assessed.

### MRI Protocol

All imaging studies were performed on a 1.5T MRI scanner (Achieva Nova; Philips Medical Systems, Best, the Netherlands) with high-performance gradients (maximum strength of 33 m·T·m^1^; slew rate of 150–180 m·T·m^-1^·s^-1^) and a 16-elements SENSE phased-array coil.

To ensure the homogenization of the intestinal activity and adequate luminal distension, all the subjects fasted for 6 hours and 1.5 L of a polyethylene glycol (PEG) solution was orally administered 30 minutes before the examination [15].

MRI protocol is summarized in Table 1.

**Table 1:** MRI sequences and parameters.

Parameters	SSFP	2D-PC FFE	3D-T1W GE
**Imaging plane**	Axial, sagittal and coronal	Axial	Coronal
**Field of view, mm** ^**2**^	Variable (320–400)	300	Variable (320–400)
**Repetition time, ms**	3.3	5.1	4.0
**Echo time, ms**	1.66	3.4	1.94
**Matrix**	220 x 186	124 x 114	188 x 187
**Section thickness, mm**	5	6	3
**Section gap, mm**	0	0	0
**Flip angle, °**	90	15	10
**Number of acquisitions**	1	1	1
**Acquisition time, s**	17	17	90–150

SSFP indicates Steady State Free Precession sequence; 2D-PC FFE: Two-Dimensional Phase-Contrast Fast-Field-Echo sequence; 3D-T1W GE: Three-Dimensional T1-Weighted Gradient-Echo sequence.

With patients in the supine position, breath-hold (SSFP) sequences were acquired to visualize the superior mesenteric vein (SMV) and artery (SMA) and the small bowel wall and to exclude any other intestinal disease.

Flow mapping was performed with a breath-hold Electrocardiography (ECG)-gated Q-Flow 2D-PC FFE sequences acquired perpendicularly to SMV and SMA [26,27], using three different levels of maximum velocity encoding (VENC): the sequence with the lowest velocity and without aliasing phenomena was considered. Bipolar velocity encoding gradients were applied along the flow direction.

To minimize bowel peristalsis, 20 mg of Scopolamine-butylbromide were administered intravenously before perfusion data acquisition (in the absence of contraindications).

MR perfusion study was performed with a breath-hold 3D T1 GE sequence in the coronal plane, after intravenous administration of 0.1mmol/kg of paramagnetic contrast material (Gadobutrol) with an automatic injector (Spectris MR, Medrad Europe, Maastricht, The Netherlands) at a rate of 2 mL/s.

### MRI Flow Analysis

**Fig 1:**
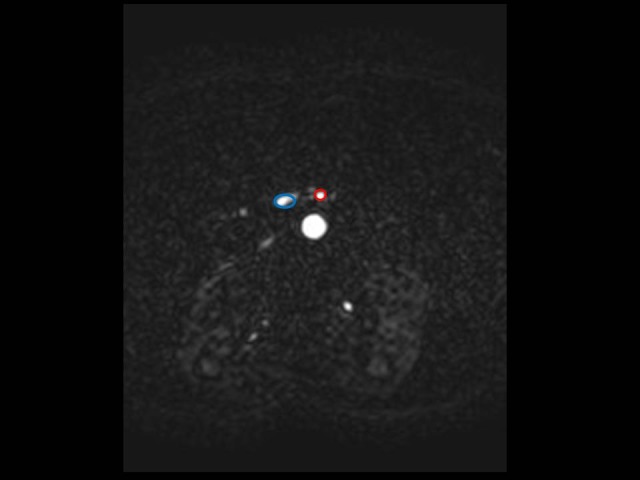
Assessment of mean flow (MF) and stroke volume (SV). In Phase-contrast sequences, elliptic ROIs were manually positioned on the superior mesenteric vein (SMV) and artery (SMA) in order to obtain curves of velocity and flow rate versus time.

Magnitude and phase MR images of the superior mesenteric artery and vein were displayed on an image-processing workstation (Extended Viewforum, Philips Medical System, Best, The Netherlands) with flow analysis package (MR Workspace 2.6.3.3). The luminal area was traced manually on the magnitude images, automatically transferred to the velocity phase images and adjusted according to the cardiac phase (Fig 1).

At various values of VENC (40, 80 and 120 cm·s^-1^), Mean Flow (MF, i.e. the quantity of blood moving through a vessel within a second, in mL·s^-1^) and Stroke Volume (SV, i.e. the volume of blood pumped out at each contraction of the heart, in mL) within each Region Of Interest (ROI) and the area of ROI (cm^2^) were determined for each cardiac frame and curves of velocity and flow rate versus time were automatically reconstructed.

### MRI Perfusion analysis

Post-processing was performed with NordicICE Software 2.3.12 (Nordic Imaging Lab AS, Bergen, Norway).

**Fig 2:**
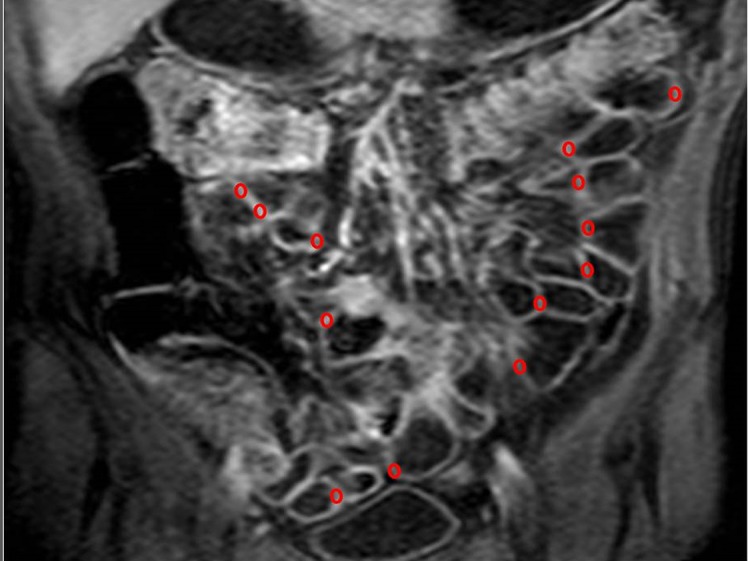
Assessment of small bowel wall perfusion. To calculate MRI perfusion parameters, AUC_60_, AUC_90_ and K^trans^, elliptic ROIs were positioned in different segments of the small bowel wall, particularly on proximal and distal jejunum and proximal, middle and distal ileum, as showed in this figure.

Elliptic ROIs (diameters of 3 x 2 mm) were manually drawn in the wall of descending duodenum, proximal and distal jejunum and proximal, middle and distal ileum (Fig 2).

To ensure the homogeneity of the data, all ROIs were placed in the segments of the small bowel mentioned above and several values for each segment were collected before finally considering their mean value.

Qualitative, semi-quantitative and quantitative maps of the parameters related to vascular permeability and intra/extra vascular volumes (based on the dynamic effect of the contrast agent) were obtained.

Areas under the signal-intensity curve from the time of injection of Gadolinium to 60 and 90 s post-injection (AUC_60_ and AUC_90_) were calculated by cubic interpolation and digital integration.

K^trans^, as two-compartment quantitative kinetic model, was calculated by setting the arterial input function on SMA [20,21].

All the measurements of flow and perfusion parameters were performed in blind by two operators (GP and FDC, respectively with 5 and 20 years of experience in abdominal MRI) who placed the ROIs and assessed measurements, blinded to all clinical data.

### Statistical analysis

Data analysis was performed by using IBM SPSS Statistics software (version 20.0; SPSS, Chicago, Ill., USA).

All parameters were checked graphically for central tendency, spread and skew.

Due to the small and independent samples of patients, data were compared with Mann-Whitney Test for independent samples.

The relationships between flow and perfusion parameters were assessed with Spearman rank correlation test.

Inter-observer agreement was evaluated with Spearman rank correlation test.

A *P* value of less than 0.05 (two-tailed testing) indicated a statistically significant difference.

The review of the manuscript was performed by F.D.C., with a 20-year post-fellowship experience in abdominal MRI.

## Results

Clinical and biochemical parameters (i.e. age, sex, body mass index, arterial blood pressure, heart rate, years from PNH diagnosis, granulocytes and red cells PNH clone percentages, concentration of Hemoglobin, serum LDH levels, need of transfusions, pain score) are listed in Table 2.

**Table 2:** Demographic and clinical features of enrolled patients.

	***Pain***						**No pain**					
**Patients**	*1*	*2*	*3*	*4*	*5*	*6*	**7**	**8**	**9**	**10**	**11**	**12**
**Sex**	*F*	*M*	*M*	*F*	*M*	*M*	**F**	**M**	**F**	**F**	**F**	**F**
**Age** *	*21*	*37*	*45*	*59*	*43*	*44*	**57**	**19**	**39**	**55**	**46**	**55**
**BMI** *	*22*	*24*	*24*	*32*	*19*	*29*	**23**	**22**	**19**	**25**	**23**	**24**
**BP, mmHg**	*120/80*	*116/63*	*125/75*	*120/90*	*122/80*	*120/80*	**130/80**	**130/80**	**100/ 70**	**130/ 87**	**110/ 80**	**120/ 80**
**HR, bpm**	*83*	*70*	*90*	*83*	*80*	*80*	**75**	**60**	**80**	**80**	**75**	**80**
**y from diagnosis**	*1*	*0*	*9*	*21*	*16*	*1*	**4**	**2**	**2**	**3**	**15**	**6**
**Clone WC** * **, %**	*77*.*0*	*70*.*0*	*99*.*7*	*46*.*0*	*85*.*7*	*99*.*0*	**31.1**	**93.0**	**77.5**	**90.0**	**53.0**	**91.5**
**Clone RC, %**	*47*.*0*	*0*.*0*	*44*.*0*	*25*.*6*	*99*.*0*	*58*.*0*	**2.7**	**65.0**	**96.6**	**90.0**	**19.0**	**26.7**
**Hb** * **, g/dl**	*12*.*0*	*7*.*9*	*8*.*7*	*9*.*6*	*13*.*8*	*9*.*5*	**10.7**	**7.7**	**12.4**	**9.1**	**13.0**	**11.4**
**LDH** * **, mU/mL**	*829*	*606*	*2456*	*1086*	*395*	*1459*	**987**	**1960**	**545**	**4023**	**672**	**1571**
**Transfusions**	*no*	*no*	*yes*	*no*	*no*	*No*	**no**	**no**	**no**	**Yes**	**no**	**Yes**
**Pain onset, y**	*2007*	*2012*	*2008*	*1990*	*1998*	*2006*						
**Pain score**	*7*	*8*	*6*	*6*	*5*	*7*						
**Pain: features**	*CWP*	*R*	*R*	*CWP*	*R*	*R*						
**Exacerbations**	*W*	*W*	*M*	*M*	*3M*	*M*						
**Duration**	*H*	*2-3D*	*2-3D*	*2-3D*	*2-3D*	*2-3D*						

SD indicates standard deviation; BMI, Body Mass Index; BP, Blood Pressure (mmHg ≈ 133.322 Pa); HR, Heart Rate; bpm, beats per minute; WC, Granulocytes; RC, red cells; Hb, Hemoglobin; LDH, Lactate Dehydrogenase; CWP, Chronic with Peaks; R, Recurrent; W, Weekly; M, Monthly; 3M, Every three months; H, Hours; 2-3D, two-three days.

* There is no significant difference between the two groups (pain vs no pain) in terms of Age: (P = 0.59), BMI (P = 0.39), PNH clone in the granulocytes (P = 0.82), LDH serum levels (P = 0.59) and Hemoglobin (P = 0.82).

All blood tests were performed at the time of MRI scan.

No significant differences were seen between the AP and NOP groups in terms of age (*P* = 0.59), BMI (*P* = 0.39), PNH clone in the granulocytes (*P* = 0.82), LDH serum levels (*P* = 0.59) and Hemoglobin (*P* = 0.82) (Table 2).

The mean DCE-MRI data both in each intestinal segment and in the whole small bowel were significantly lower in the AP than in the NOP group (Table 3).

**Table 3:** Results.

	**Patients with pain**	**Patients without pain**	***P* value**
**AUC**_**60**_ **small bowel**	84.81 ± 4.99	131.73 ± 18.47	< 0.001
**AUC**_**90**_ **small bowel**	102.33 ± 5.76	152.58 ± 23.11	< 0.001
**K** ^**trans**^ **small bowel, min** ^**-1**^	0.0346 ± 0.0019	0.0521 ± 0.0015	< 0.001
**AUC**_**60**_ **duodenum**	81.26 ± 4.61	115.34 ± 11.34	0.002
**AUC**_**90**_ **duodenum**	96.02 ± 11.42	127.86 ± 13.55	0.002
**K** ^**trans**^ **duodenum, min** ^**-1**^	0.0363 ± 0.0129	0.0511 ± 0.0094	0.093
**AUC**_**60**_ **jejunum**	90.51 ± 12.10	151.74 ± 12.52	0.002
**AUC**_**90**_ **jejunum**	107.29 ± 15.13	173.65 ± 14.32	0.002
**K** ^**trans**^ **jejunum, min** ^**-1**^	0.0350 ± 0.0067	0.0538 ± 0.0084	0.009
**AUC**_**60**_ **ileum**	82.66 ± 15.58	128.12 ± 10.46	0.002
**AUC**_**90**_ **ileum**	103.69 ± 15.62	156.24 ± 9.02	0.002
**K** ^**trans**^ **ileum, min** ^**-1**^	0.0325 ± 0.0034	0.0514 ± 0.0123	0.009
**SMV Mean Flow, mL/s**	4.67 ± 0.85	8.32 ± 2.14	0.002
**SMV Stroke Volume, mL**	3.85 ± 0.76	6.55 ± 1.57	0.015
**SMA Mean Flow, mL/s**	6.95 ± 2.61	11.2 ± 2.32	0.065
**SMA Stroke Volume, mL**	6.52 ± 2.19	8.78 ± 1.63	0.065

AUC_60_ indicates area under the curve at 60 s after contrast agent injection; AUC_90_, area under the curve at 90 s after contrast agent injection; SMV, superior mesenteric vein; SMA, superior mesenteric artery.

**Fig 3:**
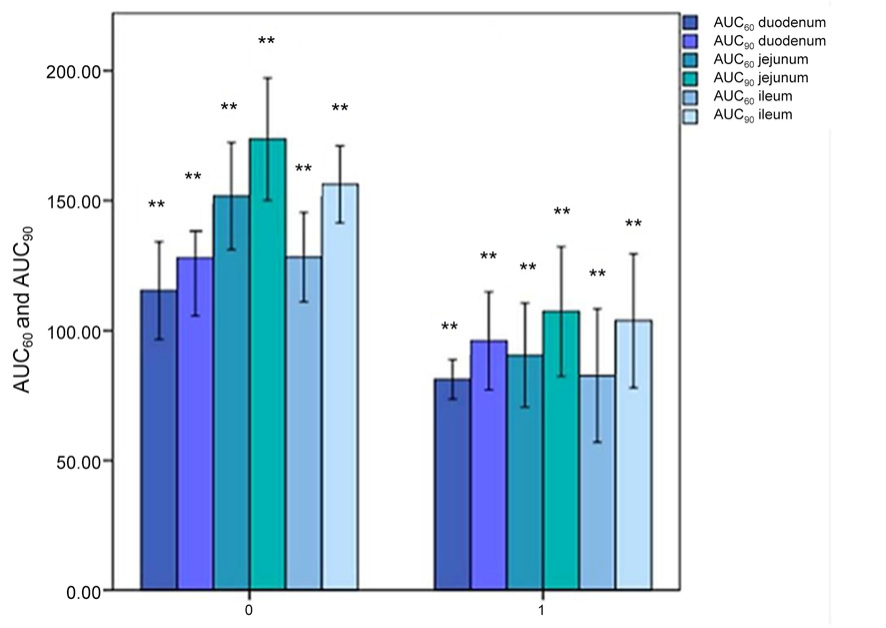
AUC_60_ and AUC_90_ in duodenum, jejunum and ileum. The horizontal axis represents the patients without (0) and with (1) abdominal pain; the vertical axis represents the AUC_60_ (darker colors) and AUC_90_ (lighter colors) values in duodenum (blue), jejunum (green) and ileum (cyan). **: *P*<.01; Errors bars: 95% of confidence interval.

In the whole small bowel AUC_60_ was 84.81 ± 4.99 in AP vs 131.73 ± 18.47 (*P* < 0.001) in NOP and AUC_90_ was 102.33 ± 5.76 vs 152.58 ± 23.11 (*P* < 0.001) (Fig 3).

**Fig 4:**
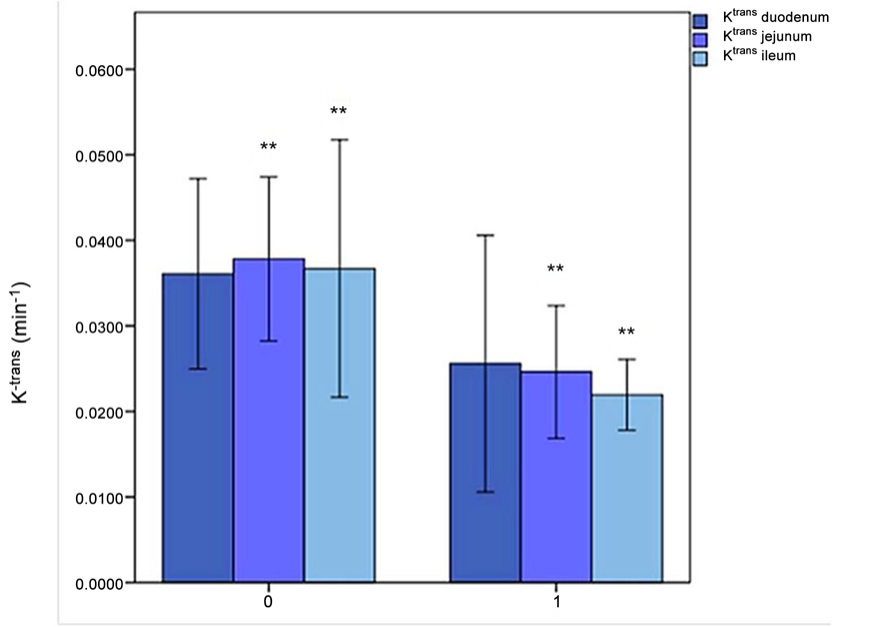
K^trans^ in duodenum, jejunum and ileum. The horizontal axis represents the PNH patients without (0) and with (1) abdominal pain; the vertical axis represents the K^trans^ values (min^-1^) in duodenum, jejunum and ileum. **: *P*<.01; Errors bars: 95% of confidence interval.

K^trans^ was 0.0346 ± 0.0019 vs 0.0521 ± 0.0015 min^-1^ (*P* < 0.001) (Fig 4).

In the duodenum AUC_60_ was 81.26 ± 4.61 in AP vs 115.34 ± 11.34 in NOP (*P* = 0.002); AUC_90_ was 96.02 ± 11.42 vs 127.86 ± 13.55 (*P* = 0.002) (Fig 3); K^trans^ was 0.0363 ± 0.0129 vs 0.0511 ± 0.0094 (*P* = 0.09) (Fig 4).

In the jejunum AUC_60_ was 90.51 ± 12.10 in AP vs 151.74 ± 12.52 in NOP (*P* = 0.002) and AUC_90_ was 107.29 ± 15.13 vs 173.65 ± 14.32 (*P* = 0.002) (Fig 3); K^trans^ was 0.0350 ± 0.0067 vs 0.0538 ± 0.0084 (*P* = 0.009) (Fig 4).

In the ileum AUC_60_ was 82.66 ± 15.58 in AP vs 128.12 ± 10.46 in NOP (*P* = 0.002) and AUC_90_ was 103.69 ± 15.62 vs 156.24 ± 9.02 (*P* = 0.002) (Fig 3); K^trans^ was 0.0325 ± 0.0034 vs 0.0514 ± 0.0123 (*P* = 0.009) (Fig 4).

Similarly, the mean blood flow MRI data (MF and SV) in the superior mesenteric artery (SMA) and vein (SMV) showed a lower flow in the AP than in the NOP group, respectively, even if a significant difference was found only in the venous compartment.

**Fig 5:**
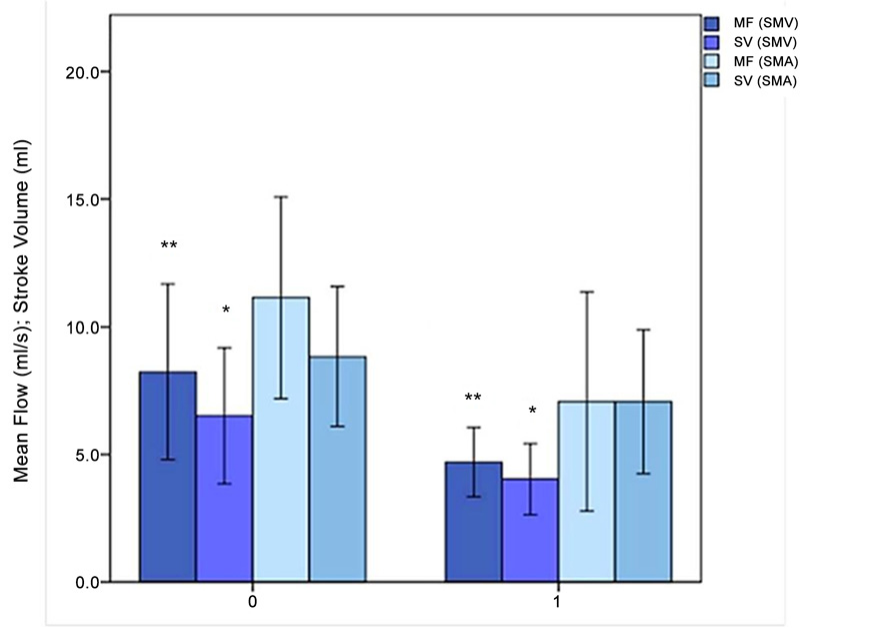
MF and SV in the mesenteric venous (SMV) and arterial (SMA) compartment. The horizontal axis represents the PNH patients without (0) and with (1) abdominal pain; the vertical axis represents the MF and SV values on superior mesenteric vein (SMV, dark blue) and artery (SMA, light blue). *: *P*<.05; **: *P*<.01; Errors bars: 95% of confidence interval.

In SMV, MF was 4.67 ± 0.85 vs 8.32 ± 2.14 mL ·s^-1^ (*P* = 0.002) and SV 3.85 ± 0.76 vs 6.55 ± 1.57 mL (*P* = 0.02) for AP and NOP, respectively (Fig 5).

In SMA, MF was 6.95 ± 2.61 vs 11.2 ± 2.32 (*P* = 0.07) and SV was 6.52 ± 2.19 vs 8.78 ± 1.63 (*P* = 0.07) for AP and NOP, respectively (Fig 5).

Inter-observer agreement was good both for perfusion and flow parameters in patients with pain (MF SMV:*ρ* = 0.943; *P* < 0.001; SV SMV:*ρ* = 0.986; *P* < 0.001; MF SMA:*ρ* = 0.943; *P* = 0.005; SV SMA: *ρ* = 0.986; *P* < 0.001; total AUC_60_: *ρ* = 0.957; *P* < 0.001; total AUC_90_: *ρ* = 0.944; *P*< 0.001; total K^trans^:*ρ* = 0.998; *P* < 0.001) and in patients without pain (MF SMV: *ρ* = 0.986; *P* < 0.001; SV SMV: *ρ* = 0.943; *P* < 0.001; MF SMA: *ρ* = 0.943; *P* < 0.001; SV SMA: *ρ* = 0.986; *P* < 0.001; total AUC_60_: *ρ* = 0.973; *P* < 0.001; total AUC_90_: *ρ* = 0.983; *P* < 0.001; total K^trans^: *ρ* = 0.991; *P* < 0.001).

**Fig 6:**
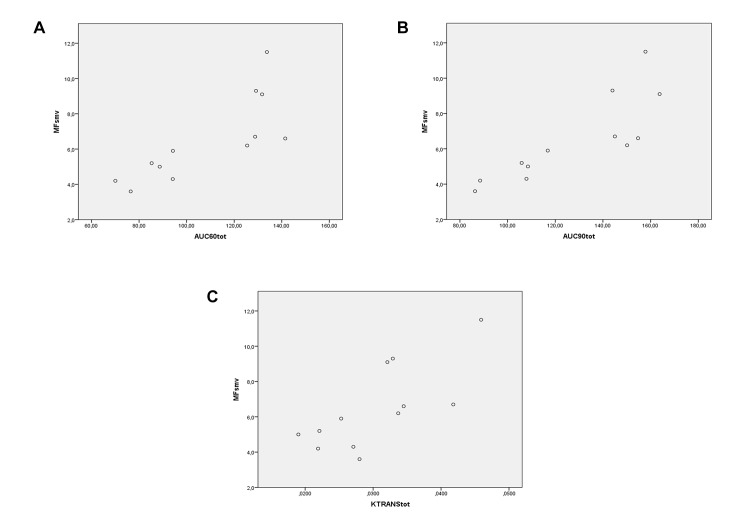
Spearman rank correlation analysis. Correlation between MRI perfusion parameters (on the horizontal axis)—AUC_60_ (A), AUC_90_ (B), and K^trans^ (C)—in the whole small bowel—of PNH patients and MF of SMV (on the vertical axis).

**Fig 7:**
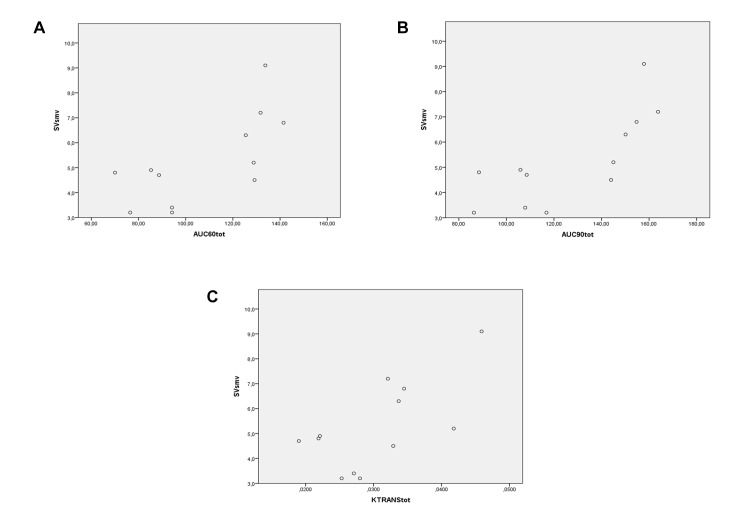
Spearman rank correlation analysis. Correlation between MRI perfusion parameters (on the horizontal axis)—AUC_60_ (A), AUC_90_ (B), and K^trans^ (C)—in the whole small bowel—of PNH patients and SV of SMV (on the vertical axis).

**Fig 8:**
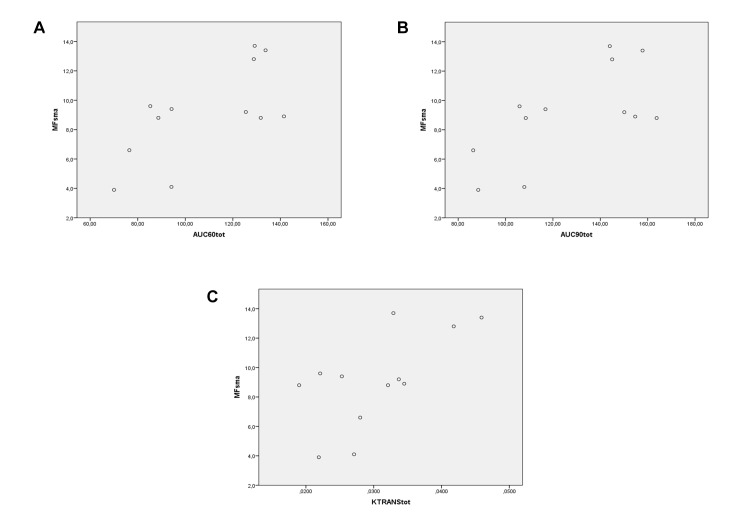
Spearman rank correlation analysis. Correlation between MRI perfusion parameters (on the horizontal axis)—AUC_60_ (A), AUC_90_ (B), and K^trans^ (C)—in the whole small bowel—of PNH patients and MF of SMA (on the vertical axis).

**Fig 9:**
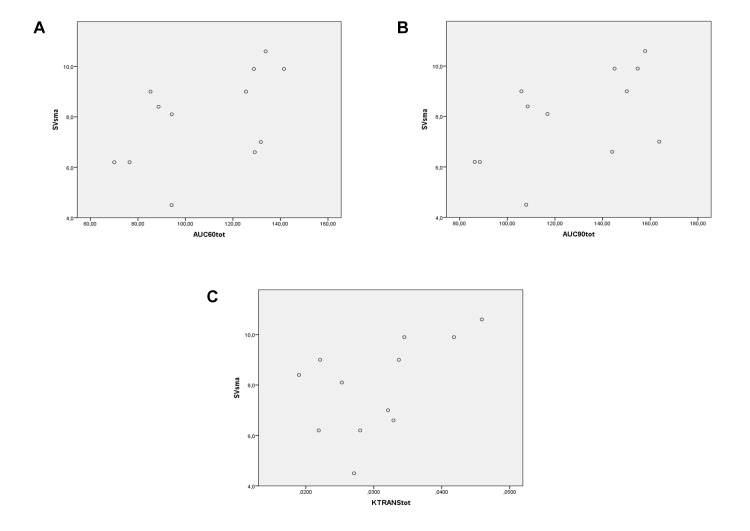
Spearman rank correlation analysis. Correlation between MRI perfusion parameters (on the horizontal axis)—AUC_60_ (A), AUC_90_ (B), and K^trans^ (C)—in the whole small bowel—of PNH patients and SV of SMA (on the vertical axis).

Regarding the correlation analysis, we obtained these results:

1. MF in SMV vs total AUC_60_:*ρ* = 0.88, *P* < 0.001 (Fig 6A); total AUC_90_:*ρ* = 0.874, *P* < 0.001 (Fig 6B); total K^trans^:*ρ* = 0.734, *P* = 0.007 (Fig 6C).
2. SV of SMV vs total AUC_60_:*ρ* = 0.644, *P* = 0.024 (Fig 7A); total AUC_90_:*ρ* = 0.774, *P* = 0.003 (Fig 7B); total K^trans^:*ρ* = 0.581, *P* = 0.047 (Fig 7C).
3. MF of SMA vs total AUC_60_:*ρ* = 0.546, *P* = 0.066 (Fig 8A); total AUC_90_:*ρ* = 0.459, *P* = 0.134 (Fig 8B); total K^trans^:*ρ* = 0.553, *P* = 0.062 (Fig 8C).
4. SV of SMA vs total AUC_60_:*ρ* = 0.580, *P* = 0.048 (Fig 9A); total AUC_90_:*ρ* = 0.608, *P* = 0.036 (Fig 9B); total K^trans^:*ρ* = 0.580, *P* = 0.048 (Fig 9C).

## Discussion

Our prospective study suggests that both small bowel blood flow and perfusion impairment might be reliable MRI markers of mesenteric ischemia in untreated PNH patients with abdominal pain.

The thrombophilic tendency in PNH patients is a multifactorial phenomenon related to a consensual activation of platelets and complement system leading to endothelial dysfunction with thrombin generation and fibrinolytic defect [3].

Unchecked complement activity has a direct effect on platelets and can initiate thrombosis, which activates the complement system triggering a vicious thrombophilic cycle until the patient develops potentially lethal major thrombotic complications [3].

Of note, while intestinal ischemia has been postulated to be the cause of recurrent bouts of abdominal pain in patients with PNH [28,29], there are few data concerning a direct ante-mortem evidence of this aspect [30].

Dolezel et al. have interestingly investigated the presence of small bowel wall thickening (suggesting recurrent ischemia) in a single case of PNH with recurrent abdominal pain using Computed Tomography (CT) and MRI [31].

Furthermore, several studies [17,19,26,27] have previously evaluated the presence of mesenteric ischemia analyzing flow and perfusion by MRI, confirming a reduction of small-bowel perfusion and mesenteric venous flow.

Our study adds to the current literature by providing initial evidence of the differences in mesenteric flow and in small bowel wall perfusion in PNH patients with and without abdominal pain.

It is known that flow characteristics of the superior mesenteric artery and vein can be qualitatively and quantitatively assessed with MR phase-contrast sequences [18,19,26,27].

As a consequence, MR flow quantification on these vessels reflects the whole small intestine blood supply [18]. Moreover, performing DCE-MRI (i.e. measuring the contrast-induced changes in tissue T1 relaxivity) allows to investigate microvessels density and capillary endothelial permeability [32,33].

In our study, we focused both on DCE semi-quantitative AUC (that represents the integrated area under the contrast medium concentration–time curve at different time points post contrast agent injection, in our case 60 and 90 s) and on the quantitative K^trans^, that reflects the two-compartment pharmacokinetic model of the contrast medium (intravascular and extra vascular components) [34].

K^trans^ is dependent on flow and permeability-surface area.

As amply demonstrated in the Literature [35,36], low values of K^trans^ indicate low permeability and/or low perfusion.

Of note, we found that in PNH patients with abdominal pain, all flow parameters were significantly lower in the venous compartment together with the AUC and K^trans^ values (especially in jejunum and ileum). In addition to this, low levels of MRI perfusion parameters in the whole small bowel were independently and strictly associated with low MF (*P* < 0.001) and SV (*P* < 0.05) values in SMV, confirming that in patients with PNH blood flow is strongly reduced in the venous district.

Conversely, we observed that the arterial compartment was less involved, as only SV of SMA, but not MF, was significantly associated with perfusion parameters (*P* < 0.05).

In order to investigate this interesting finding, it is realistic to assume that a microvascular damage occurs in PNH. In fact, the increased vascular resistance/density and the intrinsic low venous flow, might increase the time of contact/transit of pro-inflammatory mediators and reactive oxygen species along the intimal layer of the vessels.

As a result, this condition may lead to endothelial dysfunction, self-perpetuating the damage, as highlighted by some previous electron microscopy findings of capillaries with coarsely granular plasma and fibrin plugs occluding the lumen [30] and confirmed on MRI by our study (i.e. the low levels of perfusion parameters, especially K^trans^).

Moreover, nitric oxide scavenging/depletion and the burden of oxidative stress in the local vascular bed might cause constriction and spasm of the small peripheral mesenteric arterial vessels with transient ischemia and consequent reduction in the venous drainage of the mesenteric compartment.

This might lead to vascular dysfunction and microthrombosis, often associated with brisk crisis of abdominal pain, triggered by conditions that induce complement activation and the subsequent intravascular hemolysis.

We acknowledge an important limitation of our study, specifically the small number of patients: this is mainly due to the rarity of this pathology and the difficulty to recruit untreated PNH patients without any other concomitant disease.

Nevertheless, we deem that our results provide initial evidence of the importance of MRI as a reliable tool to analyze mesenteric ischemia in untreated PNH patients with abdominal pain and point out the need of larger prospective studies investigating the main purpose of this report.

## Conclusions

Small bowel blood flow and perfusion impairment seem to be early and reliable MRI markers of mesenteric ischemia in untreated PNH patients with abdominal pain.

From a clinical point of view, our initial findings could be useful to select those patients who could benefit from an early tailored therapy with Eculizumab and to monitor treatment, as assessed through flow and perfusion changes before and after therapy.
